# Supplementary material for: Investigating the influence of physiologically relevant hydrostatic pressure on CHO cell batch culture
Source: Sci Rep. 2021 Jan 8;11:162. doi: 10.1038/s41598-020-80576-8 (PMC7794228; doi:10.1038/s41598-020-80576-8)
Supplement: Supplementary file 1 — Supplementary Information 1. [file 41598_2020_80576_MOESM1_ESM.zip › S1/IgG/Feb14 0 30 60 90/SS1 Feb6 2.pdf]

Data File C:\Users\P...emStation\1\Data\TK_CHO_IgG 2019-02-14 14-17-55\032-5-ML SS1 Feb6.D
Sample Name: ML SS1 Feb6

===

Acq. Operator : SYSTEM Seq. Line : 32
Sample Operator : SYSTEM
Acq. Instrument : HPLC-0039 Location : 5
Injection Date : 2/14/2019 4:32:05 PM Inj : 1
Inj Volume : 100.000 µl
Method : C:\Users\Public\Documents\ChemStation\1\Data\TK_CHO_IgG 2019-02-14 14-17-55
\CHO_IgG_LC.M (Sequence Method)
Last changed : 2/8/2019 3:17:23 PM by SYSTEM

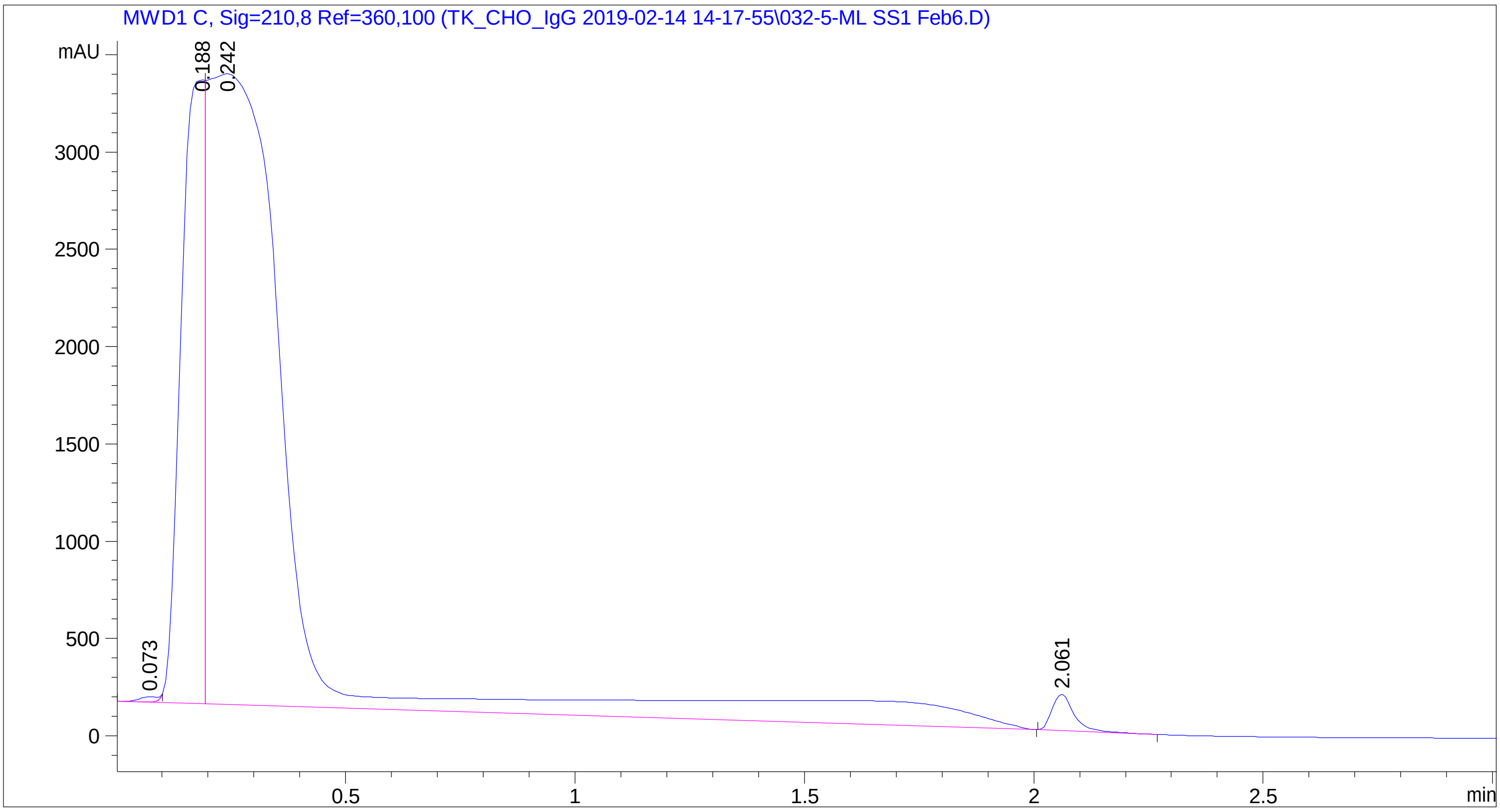

===
Area Percent Report
===

Sorted By : Signal
Multiplier : 1.0000
Dilution : 1.0000
Use Multiplier & Dilution Factor with ISTDs

Signal 1: MWD1 C, Sig=210,8 Ref=360,100

Peak #	RetTime [min]	Type	Width [min]	Area [mAU*s]	Height [mAU]	Area %
1	0.073	BV E	0.0412	73.57595	27.58522	0.1401
2	0.188	VV R	0.0560	1.10822e4	3204.85791	21.0999
3	0.242	VB	0.1866	4.07394e4	3241.47070	77.5654
4	2.061	BBA	0.0512	627.42920	186.72462	1.1946

Totals : 5.25227e4 6660.63846

===

*** End of Report ***
